# The COVID-19 Pandemic Changes the Nudging Effect of Social Information on Individuals' Blood Donation Intention

**DOI:** 10.3389/fpsyg.2021.736002

**Published:** 2021-10-26

**Authors:** Wenhua Wang, Shuaiqi Li, Jianbiao Li, Yujun Wang

**Affiliations:** ^1^China Academy of Corporate Governance, Business School, Nankai University, Tianjin, China; ^2^Institute for Study of Brain-like Economics, School of Economics, Shandong University, Jinan, China; ^3^School of Finance, Shandong University of Finance and Economics, Jinan, China; ^4^Department of Economic and Management, Nankai University Binhai College, Tianjin, China

**Keywords:** COVID-19, blood donation, nudge, social information, information content, information source

## Abstract

The positive effect of social information on nudging prosocial behavior is context dependent. Understanding how sensitive intervention outcomes are to changes in the choice context is essential for policy design, especially in times of great uncertainty, such as the current COVID-19 pandemic. The present paper explores the effectiveness of social information in changing voluntary blood donation intention in two contexts: before and after the peak of the COVID-19 pandemic in China. In addition to the dimension of context, information content and its source are also important. Using a survey administered to 1,116 participants, we conducted an intertemporal randomized-controlled experiment to systematically analyze how information can effectively nudge the intention to donate blood. Compared with content featuring blood donors' commendation information, blood users' demand information is found to have a stronger nudging effect. An official information source has a greater influence on participants' donation intention than an unofficial source. Furthermore, our analysis of two waves of experimental data (i.e., before and after the peak of the COVID-19 pandemic) shows that the COVID-19 pandemic has further enhanced the nudging effect of blood users' demand information and official information sources. These findings provide a theoretical basis and policy recommendations for relevant institutions to develop effective blood donation campaign strategies.

## Introduction

The COVID-19 pandemic poses a substantial challenge to global human well-being. Globally, it has been creating major disruptions at all levels of healthcare provision (Stanworth et al., 2020). Maintaining an adequate and consistent supply of blood to support ongoing needs is critical, as blood transfusion is essential for the operation of modern health services. Given safety and ethical concerns, non-government organizations, such as the World Health Organization, advocate donating blood, “the gift of life,” in a voluntary and unremunerated manner (World Health Organization, 2015). However, the number of active and regular blood donors is rarely able to meet the clinical demands for blood. Therefore, there is a high demand for the identification of appropriate interventions to promote voluntary and unpaid blood donation intention and behavior.

Nudges have become a popular tool for fostering prosocial behavior, and the use of nudges not restricted to situations where they make choices easier or where they exploit inertia and procrastination (Bicchieri and Dimant, 2019). One particularly promising nudge is to provide decision makers with information about others, also referred to as social information. However, social information interventions in the context of blood donation have had mixed results, with some studies demonstrating success (Sun et al., 2016; Gemelli et al., 2018; Moussaoui et al., 2019), while others either fail to detect a significant effect (Sun et al., 2019) or indicate that interventions may backfire (Goette and Tripodi, 2020). For information nudging to be effective, we must advance our understanding of the mechanisms through which information affects behavior.

According to van Teunenbroek et al. (2020), the influence of social information depends on three Ws: “where” includes social information and donors, “what” is the content of social information, and “who” is the source of social information. In this study, we incorporate the three Ws into a holistic framework to systematically analyze the nudging effect of social information on blood donation intention. The first dimension we focus on, which is also relatively ignored in the existing literature, is “where,” as the COVID-19 pandemic gives us a chance to investigate the association between changes in social context and the influence of social information. Furthermore, we construct four different kinds of social information based on the dimensions of “what” and “who” to analyze the main effects of information content and its source, as well as the interaction between the information-acting context and social information itself. Specifically, in the “what” dimension, information content is divided into blood donors' commendation information and blood users' demand information; in the “who” dimension, the information source is classified as an official source or an unofficial source.

In the context of blood donation, some studies have examined only one or two dimensions of the three Ws identified above. For example, different message content may prime different emotions among potential donors, which in turn may influence donors' perceived effectiveness of the message and their donation intentions (Song and Wen, 2019). Martín-Santana et al. (2018) emphasized the characteristics of the message source and demonstrated that spokesperson credibility is a direct antecedent of blood donation intention in radio advertising campaigns. In addition, Song and Wen (2019) just mentioned the role of contextual factors in their discussion, pointing out that differences in cultural and social norms embedded in different social contexts may lead to different perceptions of blood donation information and thus affect donation intention. It is, therefore, clear that these studies have relatively ignored the “where” and have only considered the “what” or the “who,” thereby making general comprehension quite problematic.

Our results show that blood donation intention is higher among participants who had been exposed to the blood users' demand information treatment, as compared to those who had been exposed to the blood donors' commendation information treatment. The information given by an official source also increases the participants' donation intention more than information given by an unofficial source. Furthermore, the analysis of the two waves of experimental data shows that the nudging effect of blood users' demand information is strengthened after the peak of the pandemic. Information released by official sources also exhibits a stronger nudging effect. Therefore, the COVID-19 pandemic has not only increased people's personal health- and mortality-related risk perceptions, but it may have also activated a slew of psychological mechanisms (Syropoulos and Markowitz, 2021) that changed people's perception of the same information. The findings of this study can assist researchers obtain a deeper understanding of social information and support policymakers or practitioners in choosing more effective marketing strategies for voluntary blood donation campaigns.

## Theoretical Background and Hypotheses Development

### Motivational Determinants of Blood Donation

Previous studies have shown that individuals' blood donation behaviors are often driven by three main motivations: prosocial, reciprocity, and self-image. Prosocial motivation is considered to be one of the strongest motivators for voluntary blood donation. More specifically, prosocial motivation can be labeled altruism (a desire to help other people generally) or collectivism (a desire to help members of a target group, including the donor's community and friends/family) (Bednall and Bove, 2011; Martín-Santana et al., 2020). Reciprocity is also a frequently cited motivator of donation behavior. Some donors will donate blood out of gratitude after themselves or their families have received transfusions, or in the hope that blood is available when they have a future need (Bednall and Bove, 2011). The third motivator, concern over self-image (Engel and Kurschilgen, 2020), encourages individuals to behave in a more prosocial manner in order to avoid negative judgment from others and to protect their reputations (Sénémeaud et al., 2017).

Interventions to retain existing blood donors and recruit new ones have been proposed based on existing research into donor characteristics and motivations. In their review, Godin et al. (2012) classified non-incentive interventions into four types: social interventions that manipulate altruism and egoism, reminders, foot-in-the-door or door-in-the-face techniques, and intention activation. Most of these interventions are implemented through the provision of social information, including descriptions of social impact (Moussaoui et al., 2019; Goette and Tripodi, 2020); comparisons with social norms (Xie et al., 2019); modeling (Rushton and Campbell, 1977); descriptions of a current blood shortage (Sun et al., 2016, 2019); registry invitations (Heger et al., 2020); or questionnaires asking donors to specify their donation intention to activate cognitions about blood donation (Stutzer et al., 2011). However, evidence related to the efficacy of these information interventions is mixed.

Using the theoretical framework that includes “where,” “what” and “who”(van Teunenbroek et al., 2020), the present study aims to provide a deeper understanding of how social information nudges blood donation intention. We not only study the main effects of information content and its source, but also how the information-acting context may interact with social information to shape behavioral intention.

### Influence of Information Content on Blood Donation Intention

The first independent variable manipulated between subjects is related to the dimension of “what,” namely information content: in one condition, participants are presented with blood donors' commendation information; in the other condition, blood users' demand information is given. We do so based on the assumption that people who contemplate donating blood may consider the situation from either the perspective of a potential donor or from that of the people in need of help (Hung and Wyer, 2009). The two perspectives may be fundamentally different, as the arousing content or emotional intensity of these different cues may elicits different processing patterns (Liu and Bailey, 2019).

On the one hand, an extensive body of work has demonstrated that witnessing others' prosocial actions or being provided such information can drive people to engage in similar behaviors later on (see the reviews by Jung et al., 2020). A field experiment conducted by Rushton and Campbell (1977) showed that people who observed a positive role model were more likely to donate blood, not only immediately after the exposure, but also in different settings 6 weeks later. Bruhin et al. (2020) also found strong evidence for motivational spillovers in the context of voluntary blood donations, as 40 to 44 percent of the change in an individual's propensity to donate directly spills over to their fellow tenant's propensity to donate. These spillovers generate a substantial social multiplier for policy interventions, such as phone calls reminding about the time and location of the blood drive.

Furthermore, what happens after a model's behavior can affect the degree of imitative or matching behavior exhibited by an observer. People care about how important others approve or disapprove of their performing given behaviors before they actually have respective behavioral changes (Liu and Bailey, 2020). Compared with the situation in which a model's behavior is followed by punishment or no positive reinforcement, the prosocial modeling effect can be larger when the prosocial model is rewarded by a third party (e.g., confederate, experimenter, model target) *via* social approval, gratitude, or material compensation (Jung et al., 2020). This is because the rewards may have signaled the social acceptability and desirability of the specific behavior.

On the other hand, appeals with a detailed description of the victim's plight or a picture of the beneficiary has a greater impact on participants' willingness to donate than an abstract plea for help (Hung and Wyer, 2009). Emotional reactions, such as empathic concern, can be triggered through a vivid representation of victims and often increase with the severity of the situation (Cialdini et al., 1997). This enhanced emotional involvement is fundamental for prosocial attitudes (Haidt, 2001), judgments and decisions (Slovic et al., 2002), particularly decision making in helping situations (Batson, 2011), where feeling more is assumed to be related to helping more. Laboratory experiments have shown that an identifiable victim is more likely to evoke empathy and incentivize people to donate (Kogut and Ritov, 2005). By randomizing advertising content in their field experiments, Sudhir et al. (2016) also found a significant impact that is consistent with the identified victim effect on the number of donors and amounts donated. These studies imply that narratives about the suffering of specified others may foster a desire to help.

Based on a dual deliberative (cognitive) and affective (emotional) process model of cognition (Kahneman, 2011), we propose that blood donors' commendation information nudges donation intention by activating the deliberative system (System 2), whereas blood users' demand information invokes the affective system (System 1). Specifically, the influence of blood donors' commendation information could be supported by the cultural learning account of prosocial behavior (Jung et al., 2020), which proposes that human prosociality is a direct product of social learning (Chudek and Henrich, 2011). The presence of others displaying prosocial behavior may increase norm salience or change individuals' norm perception (Goeschl et al., 2018). The universal tendency for people to rely on social norms when making prosocial decisions subsequently results in helping outcomes. Furthermore, communication that the prosocial model received a reward for helping also makes people aware that models' behaviors are encouraged by society, thereby providing an expectation of a similar social reward for mimicking their behavior (Morgenroth et al., 2015). It can be seen that blood donors' commendation information can stimulate potential donors to rethink (the relevant norms, ideals and duties in) the situation at hand (Engelen et al., 2018) and change their subjective goal expectations (Morgenroth et al., 2015). These updated thoughts, in combination with their own social experiences, promote subsequent willingness to voluntarily donate blood. This process demands cognitive investments and reflective reasoning, corresponding to the activation of System 2 (Lin et al., 2017).

In contrast, the emotional reactions associated with an urge to relieve the suffering of someone else, elicited by blood users' demand information, is fast and spontaneous (Bergh and Reinstein, 2020). The literature indicates that people are prosocial and cooperative when they make more spontaneous decisions (e.g., Rand et al., 2012; Rand, 2016). Such intuitive decision-making is distinctly associated with the operation of System 1. Systems 1 and 2 differ in the extent to which representations are accessible (Kahneman, 2003) and the effort with which particular mental contents explicitly come to mind (Brocas and Carrillo, 2014). Emotional decisions are made quicker and easier, as Kahneman (2003) argues in his theory that System 1 is the automatic system. Information processing can be facilitated by allowing affective reactions to be accessed more quickly (Johnson et al., 2012). System 2, in contrast, is commonly described as deliberate, analytical, controlled and effortful (Kahneman, 2011; Evans and Stanovich, 2013). Deliberative judgments emanating from System 2 require cognitive resources, such as working memory, attention, and self-control (Boureau et al., 2015), to play the part of monitor and intervener (Grayot, 2020). The involvement of these cognitive resources, especially self-control related resources, may suppress the potency of external information intervention (Janssen et al., 2010), thereby weakening information-based choices (Boureau et al., 2015). Therefore, we hypothesize the following:

**Hypothesis 1**. Relative to blood donors' commendation information, blood users' demand information has a stronger nudging effect on an individual's blood donation intention.

### Influence of Information Source on Blood Donation Intention

When people receive information, it is important who provides it (van Teunenbroek et al., 2020). To examine the effect of information source, we added statements showing different subject attributes to the beginning of the donors' or users' material, including official, and unofficial sources.

Kim (2010) argues that a credible source of information is most frequently quoted by the respondents (general public in the age range of 20 to 30) as an important element for influencing them to perceive the information as useful. High credibility sources, compared with low credibility ones, are likely to change attitudes in the direction of the advocated position (Hovland and Weiss, 1951; Kumkale et al., 2010). Behavior can also be facilitated by perceptions of the source's credibility (Cheung et al., 2009). Public health messages have been shown to be more effective in changing behavior during pandemic when trusted voices are enlisted to deliver the message (Van Bavel et al., 2020).

In the context of blood donation appeals, the credibility of information also has a strong positive impact on receivers' intentions (Fonte et al., 2017; Martín-Santana et al., 2018). An authoritative image is a main contributor and predictor of the information being perceived as credible by young adults (Rieh, 2010). The “authority effect” is a powerful social influence principle frequently used in advertising to increase compliance (Jung and Kellaris, 2006). Thus, the following hypothesis is formulated:

**Hypothesis 2**. Compared with unofficial sources, when the information comes from an official source, social information has a stronger nudging effect on an individual's blood donation intention.

### Changes in Social Context Brought by the COVID-19 Pandemic

As for the “where” dimension, the current literature shows that cultural characteristics, societal differences and some other social context-related factors may influence individuals' donation intention (Li et al., 2021). Depending on the distribution of context, the aggregate effect of a given piece of information might be markedly different, especially in times of great uncertainty, such as the current COVID-19 pandemic.

Firstly, the widespread collective action and cooperation that occurred during the peak of the COVID-19 pandemic (Syropoulos and Markowitz, 2021) may make society as a whole more collectivistic. It has been suggested that collectivism, as an important cultural value, can affect a person's sensitivity to prosocial norms (Jung et al., 2020). Secondly, plenty of news reporting appearing during the pandemic about ordinary people as role models has made individuals realize that “ordinary people can be true heroes,” thereby decreasing the psychological distance between people and these role models (Wessler and Hansen, 2017) and raising the desirability of obtaining social rewards for imitating their behavior. Thus, the effectiveness of blood donors' commendation information may have increased after the peak of the pandemic.

Secondly, the increasing severity of the pandemic has forced people to focus on the suffering and misfortune of others. People are increasingly capable of feeling and understanding other people's situations and emotions (Jin et al., 2020). Researchers have found that people with a stronger sense of empathy are more likely to be motivated to engage in prosocial behaviors, such as donating to charitable projects (Telle and Pfister, 2012; Murillo et al., 2016). Therefore, the effectiveness of information about blood users' demand may have also increased.

However, when people feel threatened by a range of emergencies and disasters, they may pay more attention to negative information, such as the suffering of others, than positive or neutral information (Van Bavel et al., 2020) and are more likely to be emotionally driven to make decisions. Therefore, we hypothesize the following:

**Hypothesis 3a**. Compared with information about blood donors' commendation, the context of the COVID-19 pandemic has enhanced the nudging effect of information about blood users' demand.

On the other hand, the COVID-19 pandemic has already seen a rise in fake news and misinformation. In this context, it is difficult for the public to distinguish scientific evidence and facts from less reliable sources of information (Van Bavel et al., 2020). It has also been suggested that the perceived threat triggered by the pandemic may lead people to display increased trust toward authorities such as governments (Yam et al., 2020) because doing so reduces uncertainty. Therefore, the following hypothesis is proposed:

**Hypothesis 3b**. Compared with unofficial information sources, the context of the COVID-19 pandemic has enhanced the nudging effect of the information released by official sources.

## Materials and Methods

### Experiment Design and Measures

The experiment used a 2 (information content: blood donors' commendation information vs. blood users' demand information) × 2 (information source: official sources vs. unofficial sources) × 2 (context: before vs. after the outbreak of the COVID-19 pandemic) between-subject design to explore how the three dimensions influence the effectiveness of social information on nudging blood donation intention. Content and source are reflected in the presentation of the information itself. In the condition of blood donors' commendation information, the experimental materials were adapted from the document No. 42 (2018) issued by the National Health Commission of China, while information about the blood users' demand was based on the real events of the Jiuzhaigou Earthquake in August 2017. For source manipulation, we added statements showing different subject attributes to the beginning of the donors' or users' material. The impact of information context was studied by conducting the same experiment twice: once in January 2019 and once February 2021, with the utilization of the COVID-19 pandemic. In China, the COVID-19 pandemic had been significantly abated by February 2021. Additional details of the experimental materials are provided in Table 1.

**Table 1:** The detailed experimental materials.

**Content**	**Source**	**Details**
Blood donors' commendation information	Official subjects	**Official documents issued by National Health Commission “The decision on honoring the winners of the 2016–2017 Gold Award for Voluntary and other award winners' decisions” (China National Health Medical Institute (2018) No. 42) announced:** The National Health Commission, the Red Cross Society of China and the Health Bureau of the Logistical Support Department of the Central Military Commission have decided: 71,123 comrades, including Wang Liyou, who made outstanding achievements in blood donation work during 2016–2017, were awarded the “Gold Award for Voluntary Blood Donation;” 84,991 comrades, including Jia Chengzhen were awarded the “Silver Award for Voluntary Blood Donation;” 235,855 comrades, including Wanghui were awarded the “Bronze award for Voluntary Blood Donation;” 202 comrades, including Ji Hongwen were awarded the “Catalyst Award for Voluntary Blood Donation;” 9,390 comrades, including Liu Lirong were awarded the “Service Award for Voluntary Blood Donation.”
	Unofficial subjects	**A WeChat group administrator posted the following in his group:** 71,123 comrades, including Wang Liyou, who made outstanding achievements in blood donation work during 2016–2017, were awarded the “Gold Award for Voluntary Blood Donation;” 84,991 comrades, including Jia Chengzhen were awarded the “Silver Award for Voluntary Blood Donation;” 235,855 comrades, including Wanghui were awarded the “Bronze award for Voluntary Blood Donation;” 202 comrades, including Ji Hongwen were awarded the “Catalyst Award for Voluntary Blood Donation;” 9,390 comrades, including Liu Lirong were awarded the “Service Award for Voluntary Blood Donation.”“
Blood users' demand information	Official subjects	**Jiuzhaigou Tourism Management Department issued the following news:** An earthquake struck Jiuzhaigou on August 8. A 37-year-old man surnamed Lv, his wife surnamed Ye and their daughter were hit by a rock. Blood gushed from the wounded, and a puddle of blood suddenly appeared on the ground. The face of the wounded turned from red to yellow, from yellow to white. The wounded was dying and their body temperature dropping. They were in urgent need of blood transfusion.
	Unofficial subjects	**A visitor posted a message in the WeChat group:** An earthquake struck Jiuzhaigou on August 8. A 37-year-old man surnamed Lv, his wife surnamed Ye and their daughter were hit by a rock. Blood gushed from the wounded, and a puddle of blood suddenly appeared on the ground. The face of the wounded turned from red to yellow, from yellow to white. The wounded was dying and their body temperature dropping. They were in urgent need of blood transfusion.

The dependent variable was the voluntary blood donation intention. It has been specified that an individual's intention to perform a behavior is the most proximal determinant of that behavior (Ajzen, 1991). Participants reported their intentions on a 5-point, 1-item Likert scale (i.e., “Would you like to donate blood after seeing this information”), ranging from 1 (“very strongly unwilling”) to 5 (“very strongly willing”). Other control variables used include participants' demographic information, gender, age, major, political affiliation, household per capita monthly income, and past experience of blood donation.

### Participants

Young adults represent the largest proportion of new and current blood donors; this group is essential for the maintenance of a sufficient and sustainable future donor base (Russell-Bennett et al., 2015). We selected undergraduate and graduate students as the target sample. The survey experiment was conducted on a digital online platform called “Wenjuanxing” in Mainland China, which provides functions equivalent to Amazon Mechanical Turk. A total of 1,185 participants^1^ were recruited through WeChat to participate in the online experiment. Among these participants, 886 participated in January 2019, and 299 participated in February 2021. Surveys at both timepoints lasted ~10 min, and participants received $0.30–0.40 as remuneration for their participation.

Out of all participants, 79 were excluded from the analysis due to incomplete information or obvious errors in their responses. In sum, 94.18% of the respondents (1,116 out of 1,185) were considered for the statistical analyses. In the experiment conducted in January 2019, 212 participants engaged in the *blood donors' commendation information, official source condition*, 209 engaged in the *blood donors' commendation information, unofficial source condition*, 201 engaged in the *blood users' demand information, official source condition*, and 209 engaged in the *blood users' demand information, unofficial source condition*. In contrast, in the experiment conducted in February 2021, there were 72, 68, 73 and 72 participants engaged in the above four conditions, respectively.

### Statistical Analyses

Descriptive analyses were conducted to describe the demographic characteristics. χ^2^-tests were used to test the null hypothesis of perfect randomization in case of binary variables, and Kruskal-Wallis tests in case of interval variables.

A 2 × 2 × 2 ANOVA was conducted to preliminarily test the hypothesis. Then, taking control variables into account, we performed moderating effect test by using PROCESS Macro (extension in SPSS) by Hayes (2013) to further check whether the blood donation intention elicited by different information was moderated by the COVID-19 pandemic. All data were analyzed by SPSS version 22.0.

## Results and Discussion

### Demographic Characteristics

We initially verified the comparability of the different conditions and periods. The results showed no significant differences in the sociodemographic characteristics of participants among the different conditions before and after the peak of the pandemic. Table 2 provides detailed summary statistics of the characteristics of the overall sample and the non-parametric test results of the eight sub-samples.

**Table 2:** Demographic characteristics of the sample and non-parametric test results.

**Variables**	**Category**	**Number**	**Percentage**	***χ^2^***	***Asymp. Sig*.**
Gender	Female	654	58.60%	4.414	0.731
	Male	462	41.40%		
Age	17–25	970	86.92%	4.774	0.687
	26–35	138	12.37%		
	36–52	8	0.72%		
Major	Humanities and Social Sciences	679	60.84%	7.588	0.370
	Science and Engineering	437	39.16%		
Only child	Yes	578	51.79%	11.714	0.110
	No	538	48.21%		
Polity	Mass	765	68.55%	11.032	0.137
	Communist Party	351	31.45%		
Income	<2,000 RMB	155	13.89%	11.785	0.108
	2,001–4,000 RMB	309	27.69%		
	4,001–6,000 RMB	283	25.36%		
	6,001–8,000 RMB	143	12.81%		
	More than 8,000 RMB	226	20.25%		
Experience	0	772	69.18%	5.217	0.633
	1	216	19.35%		
	2 times or more	128	11.47%		

### Hypothesis Testing

The mean and standard deviation data for blood donation intentions under different conditions are shown in Table 3.

**Table 3:** Results for the effects of social information on blood donation intention.

**Results**	**Context**	**Content**	**Source**	**Observations**	**Mean (SD.)**
1	Before the outbreak of pandemic	Blood donors' commendation information	Official	212	2.873 (1.276)
2			Unofficial	209	2.722 (1.217)
3		Blood users' demand information	Official	201	3.766 (1.312)
4			Unofficial	209	3.584 (1.409)
5	After the outbreak of pandemic	Blood donors' commendation information	Official	72	3.014 (1.284)
6			Unofficial	68	2.338 (1.045)
7		Blood users' demand information	Official	73	4.289 (0.889)
8			Unofficial	72	3.736 (1.199)

First, a 2 × 2 × 2 ANOVA was conducted to test the hypotheses 1 and 2. The results indicate that participants who were presented with information about blood users' demand were more willing to donate blood (*M* = 3.763) than those who were presented with the information about blood donors' commendation (*M* = 2.770)^2^, *F*_[1,1108]_ = 163.738, *p* < 0.0005, η^2^ = 0.129. Participants who read the information from official sources expressed a greater willingness to donate blood (*M* = 3.398) than those in the unofficial sources condition (*M* = 3.129)^3^, *F*_[1,1108]_ = 20.332, *p* < 0.0005, η^2^ = 0.018. While the main effect of context did not approach significance (*F*_[1,1108]_ = 1.551, *p* = 0.213, η^2^ = 0.001; *M* = 3.361 after the outbreak of the pandemic and *M* = 3.229 before the outbreak of the pandemic), the interaction between the information content and context (*F*_[1,1108]_ = 7.025, *p* = 0.008, η^2^ = 0.006), as well as the interaction between the information source and context (*F*_[1,1108]_ = 6.688, *p* = 0.010, η^2^ = 0.006) were significant. Besides, the two-way interaction between the information content and source (*F*_[1,1108]_ = 0.070, *p* = 0.791, η^2^ < 0.0005) and the three-way interaction (*F*_[1,1108]_ = 0.204, *p* = 0.651, η^2^ = < 0.0005) are both non-significant. The results are shown in Table 4.

**Table 4:** Analysis of variance results for the effect of social information on blood donation intention.

**Source**	**Type III SS**	***df***	***MS***	***F***	***p***	** $\eta_{p}^{2}$ **
Content	259.707	1	259.707	163.738	<0.001	0.129
Source	32.249	1	32.249	20.332	<0.001	0.018
Context	2.461	1	2.461	1.551	0.213	0.001
Content × Source	0.112	1	0.112	0.070	0.791	<0.001
Content × Context	11.143	1	11.143	7.025	0.008	0.006
Source × Context	10.609	1	10.609	6.688	0.010	0.006
Content × Source × Context	0.324	1	0.324	0.204	0.651	<0.001
Error	1,757.412	1108	1.586			
Total	13,964.000	1116				
Corrected total	2„078.548	1115				

Thus, the following conclusions can be made: (1) relative to blood donors' commendation information, blood users' demand information has a stronger nudging effect on an individual's blood donation intention; (2) compared with unofficial sources, when the information source is an official source, social information has a stronger nudging effect on an individual's blood donation intention; (3) the context of the COVID-19 pandemic shows no significant influence on blood donation intention. Hypothesis 1 and 2 are supported.

Second, we conducted a moderated regression analysis to further verify the nudging effect of social information on the blood donation intention taking other control variables into account. Results are presented in Table 5. As can be seen from Table 5, Model 1 results show that the hypothesized Context × Content interaction is significant such that the changes in the social environment brought about by the COVID-19 pandemic strengthened the negative relationship between the information content and blood donation intention. Blood users' demand information could stimulate participants' willingness to donate blood more than blood donors' commendation information, especially after the peak of the COVID-19. Furthermore, the coefficient of the interaction term between the context and information source in Model 2 is significantly positive, which means that compared with unofficial information sources, the nudging effect of the information released by official sources are further enhanced after the peak of the COVID-19 pandemic.^4^ These findings illustrate that the efficacy of social information strategies can in fact depend upon the content and source, especially after the COVID-19 pandemic. Hypothesis 3a and 3b are accepted.

**Table 5:** Moderation analysis.

	***Coefficient***	***SE***	***t***	***Significance(p)***	**LLCI**	**ULCI**
**Moderation Model 1 (*****Dependent*** **blood donation intention)**						
Content	−0.857	0.084	−9.985	<0.0005	−1.024	−0.688
Context	0.341	0.120	2.855	0.004	0.107	0.576
Content × Context	−0.398	0.170	−2.345	0.019	−0.731	−0.065
Conditional Effects	−0.062	0.080	−0.769	0.442	−0.219	0.096
Before the peak of pandemic	−0.856	0.086	−9.985	<0.0005	−1.025	−0.688
After the peak of pandemic	−1.255	0.147	−8.559	<0.0005	−1.542	−0.967
Demographic controls	Yes					
**Moderation Model 2 (*****Dependent*** **blood donation intention)**						
Source	0.176	0.092	1.917	0.055	−0.004	0.355
Context	−0.051	0.129	−0.396	0.692	−0.305	0.202
Source × Context	0.413	0.181	2.282	0.022	0.059	0.768
Conditional Effects						
Before the peak of pandemic	0.176	0.092	1.917	0.056	−0.0041	0.3554
After the peak of pandemic	0.589	0.156	3.767	<0.0005	0.282	0.896
Demographic controls	Yes					

**Figure 1:**
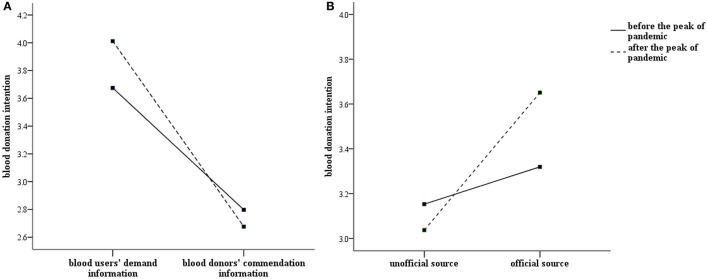
Interaction effects of information content and pandemic on blood donation intention **(A)**; Interaction effects of information source and pandemic on blood donation intention **(B)**.

Figure 1 presents a graphical depiction of the interaction. The negative relationship between information content and blood donation intention is stronger after the peak of the COVID-19 pandemic. There is no significant difference in donation intention in response to donors' commendation information before and after the outbreak of the COVID-19 (*p* = 0.382, two-sided Mann Whitney tests, the same as below). But the nudging effect of blood users' demand information is significantly strengthened after the COVID-19, compared with pre-pandemic (*p* = 0.040). At the same time, people tend to exhibit higher donation intention in response to information released by official sources after the peak of COVID-19 than before (*p* = 0.009), while unofficial information shows no significant inter-temporal effect (*p* = 0.452).

## Discussion

The main purpose of this study is to explore the nudging effect of social information on blood donation intention in two contexts: before and after the peak of the COVID-19 pandemic. Based on the theoretical framework developed by van Teunenbroek et al. (2020), we focus on the three dimensions on which the effectiveness of social information depends, namely, the information-acting context (where), the information content (what) and the information source (who). By exploiting a 2 × 2 × 2 between-subject design survey experiment, we study not only the main effects of information content and its source, but also how the information-acting context may interact with social information.

The results show a significant main effect of information content on blood donation intention, suggesting that relative to blood donors' commendation information, blood users' demand information is a more effective strategy for increasing intention toward blood donation. This is consistent with those of recent studies, which indicated that people considered other-focused arguments to be more persuasive (Luttrell and Petty, 2021). Decisions to donate are informed by both rational and emotional processes (Dickert et al., 2011), including cognitive factors such as moral judgment and social learning, and rather affective factors such as empathy (Christner et al., 2020). As the blood donors' commendation information affects the intention by triggering deliberative judgments generated by System 2, which requires cognitive resources, its nudging effect is weaker than the description of the victim's urgent needs. This finding thus highlights the importance of the emotional appeals in promoting prosocial behavior, especially blood donation behavior.

In terms of “who” dimension, we demonstrate that social information released by official sources has a stronger nudging effect. Previous study has unearthed a number of important factors impacting on donation decisions, such as the perceived credibility of the charitable organization, organizational accountability, and organizational commitment (Zagefka and James, 2015). Martins et al. (2019) also pointed that credibility is strongest predictor of the acceptance of messaging that can lead to behavior change. In China, social media suffer from the problems of information overload and prevalence of misinformation (Gao et al., 2020). Evidence shows that channels endorsed by officials were perceived to be highly credible among Chinese people (Zhang et al., 2014). Trust in official information sources is likely to increase acceptance of their proposition (Chen et al., 2018). Acceptance of information would further increase the tendency to adhere to these advocated behaviors.

Furthermore, the analysis of two waves of experimental data indicates that the nudging effect of blood users' demand information has been strengthened after the peak of the COVID-19 pandemic. An alternative explanation for such effects might be related to a slew of psychological mechanisms activated by the COVID-19 pandemic. The pandemic can be regarded as a threat to one's survival, which might activate the sense of a “common fate” and thereby increase empathy as a motivation of increasing other's welfare (Christner et al., 2020). The shared experience of a global threat could amplify people's need and thereby attune people to other's well-being. Such underlying psychological changes are likely to change responsiveness to the blood users' demand information, as the decision on whether to donate is related to general psychological state (Dickert et al., 2011). By contrast, the prosocial modeling effect of blood donors' commendation information largely depends on cognitive factors, which involve deliberative reflection. It is suggested that people do not behave differently in response to contextual cues when investing enough cognitive resources to make a decision on whether to donate (Shi et al., 2020). Instead, they displayed a stable tendency for keeping their original decision. So, the pandemic has significantly strengthened the nudging effect of users' demand information, while has no significant effect on the responsiveness to donors' commendation information.

In addition, information released by official sources also exhibits a stronger nudging effect after the peak of the COVID-19. With the impact of uncertainty brought by the pandemic, the perceived credibility of various sources of information also varies greatly in the eyes of the public. Compared with the long-standing, general trust in government which has been shaped by various social and cultural factors, the specific aspect of trust in authorities during crises, such as the COVID-19 pandemic, could be more dynamic. It has been found that the pandemic itself both relies on and may change the extent to which the public trust in government or other organizations (Van Bavel et al., 2020). Consistent with existing research results, sudden crisis situations often result in an increase in support and trust in government caused by a “rally-round-the-flag” effect (Yam et al., 2020). Research from China during the COVID-19 pandemic also revealed that public showed a higher level of perceived credibility in governments than the usual levels documented in general social surveys (Wu et al., 2021), perhaps due to effective implementation of anti-COVID policies official media propaganda, and public's expectation (Su et al., 2021). As information is thought to be more credible when they are issued by trustworthy organizations (Rifon et al., 2004), such enhanced trust in official organizations could amplify public's compliance with social policies that rely on their behavioral responses (San Lau et al., 2020).

### Theoretical Contributions

This study contributes to the relevant research on how to nudge blood donation behavior from the perspective of social information. Previous studies have focused on the nudging effects of information methods, such as sending reminders, providing feedback, and strengthening social norms (Sun et al., 2019; Goette and Tripodi, 2020; e.g., Fosgaard et al., 2020). As for social information itself, existing literature recommends developing differentiated strategies only on the basis of subdividing blood donor types (Zhou et al., 2012), without establishing a holistic framework to systematically analyze how information can effectively nudge the intention to donate blood. This study uses a survey experiment to quantitatively study the influence of the three Ws of social information on individuals' blood donation intention, which complements the current research on the nudging mechanism of blood donation intention and behavior.

Our study also adds to the nascent but exploding literature on the COVID-19 pandemic. To cope with the large-scale challenges and alleviate the negative consequences of the pandemic, it is of great significance to understand how people might react to different information interventions. The importance of finding efficient information is clear, as such information represents an easy and potentially scalable intervention; it can be texted by phone or spread on social media in a low-cost way. Our results suggest that information with a detailed description of the victim's plight and with identification of the official source can be most effective in nudging individuals' blood donation intention, especially in times of great uncertainty like the current COVID-19 pandemic.

### Managerial Implications

From the present study, a series of practical conclusions can be drawn that are particularly relevant for blood transfusion centers when managing their communication strategies. To encourage people to donate blood, the appeal for voluntary blood donation should clarify the critical situation of blood users. Detailed narratives of the urgent needs of victims can immediately make the public have a strong empathic response. Some narrative techniques need to be skillfully used to stimulate individual empathy to the greatest extent. For example, the display form of social information should not be limited to paper materials. Videos and other forms of publicity can also be used so that the public can truly experience the crisis situation in which blood users find themselves. We also suggest that more emotive words be used to elicit a high level of empathy from the public, so as to nudge their intention to donate blood.

Second, the results of this study show that individuals are more willing to donate blood when the information is released by official sources, whether it is information about blood donors' commendation or blood users' demand. Therefore, in their blood donation campaigns, official organizations should take full advantage of their brand's image and perceived authority to promote blood donation more efficiently. For example, markers that indicate the official attributes of an information source should be highlighted.

### Limitations and Future Research

Firstly, the main limitation of this study refers to the population under study (undergraduate and graduate students). Considering the intergenerational differences between individuals of different ages, their attitudes toward things may differ. It would be valuable to investigate our question in a more diverse sample. Exploring the heterogeneous impact of nudges on people, such as some of the most vulnerable groups in the pandemic, rather than the average effect collapsing across general public (e.g., Mrkva et al., 2021) would also be worthy of attention. Secondly, the measurement of blood donation intention in the present study is measured by the one-item Likert scale, “Would you like to donate blood after seeing this information.” Although individuals' blood donation intention is positively correlated with actual behavior (Ferguson and Bibby, 2002), it is still necessary to use field experiments to validate our findings. Furthermore, the question how nudging effect of such information may change at different time points or under different conditions is highly interesting, as the COVID-19 pandemic is a worldwide phenomenon and countries react differently. It is worthy to cross-country validate our findings and to explore how long the enhanced nudging effect the specific information would last in later stages of the pandemic.

## Conclusion

How to nudge voluntary and unpaid blood donation intention by exploiting social information is of great significance, especially in the midst of a global pandemic. Our results suggest that relative to blood donors' commendation information, blood users' demand information has a stronger nudging effect, social information released by official sources has a stronger nudging effect than unofficial information. And the nudging effect of blood users' demand information and information released by official sources both have been strengthened after the peak of the COVID-19 pandemic.

## Data Availability Statement

The raw data supporting the conclusions of this article will be made available by the authors, without undue reservation.

## Ethics Statement

The studies involving human participants were reviewed and approved by the Ethics Committee of Nankai University. Written informed consent to participate in this study was provided by the participants' legal guardian/next of kin.

## Author Contributions

WW, JL, and SL conceived and designed the experiments. WW, JL, and YW performed the experiments. WW analyzed the data. WW, SL, and JL contributed to the writing and revision of the manuscript. All authors contributed to the article and approved the submitted version.

## Funding

This work was supported by the National Social Science Foundation of China [Grant Numbers 20AZD044 and 18BDJ084], the National Natural Science Fund of China [Grant Number 71673152], Major Research Project of Humanities and Social Sciences of Shandong University [NO. 21RWZD15], Taishan Scholar Program of Shandong Province [NO. tsqn201909013], and National College Students' Innovation and Entrepreneurship Training Program [NO. 202113663004].

## Conflict of Interest

The authors declare that the research was conducted in the absence of any commercial or financial relationships that could be construed as a potential conflict of interest.

## Publisher's Note

All claims expressed in this article are solely those of the authors and do not necessarily represent those of their affiliated organizations, or those of the publisher, the editors and the reviewers. Any product that may be evaluated in this article, or claim that may be made by its manufacturer, is not guaranteed or endorsed by the publisher.

## Footnotes

^1^We conducted a power analysis in G^*^Power 3.1 (Faul et al., 2007) for a repeated-measures ANOVA with the following parameters: 8 groups (2 × 2 × 2 between-subject design), a type-I error level of α = 0.05, a moderate effect size of f = 0.25, a power of 0.9 and a correlation among rep measures of *r* = 0.5. This power analysis revealed that the minimum sample size in the present study is *n* = 208.

^2^The M reported here is a composite of the data from both periods before and after the pandemic.

^3^The same as above.

^4^As the individual characteristics is not the focus of this paper, we briefly report some findings for reference. Those who are members of the Communist Party are significantly more likely to donate blood. Those with siblings have a higher willingness to donate blood than only children, which is consistent with the findings of Cameron et al. (2013), who found that only children in China have a relatively lower sense of responsibility. Previous blood donations strongly predict one's propensity to donate, which is consistent with the findings of many other studies (Beerli-Palacio and Martín-Santana, 2009; Bednall and Bove, 2011).
